# Informal Allopathic Provider Knowledge and Practice Regarding Hypertension in Urban and Rural Bangladesh

**DOI:** 10.1371/journal.pone.0048056

**Published:** 2012-10-25

**Authors:** John Parr, Wietze Lindeboom, Masuma Khanam, James Sanders, Tracey Pérez Koehlmoos

**Affiliations:** 1 International Center for Diarrheal Disease Research, Bangladesh, Health Systems and Infectious Disease Division, Mohakali, Dhaka, Bangladesh; 2 Family Medicine Residency, Contra Costa County Regional Medical Center, Martinez, California, United States of America; 3 Department of Family Medicine, Medical College of Wisconsin, Milwaukee, Wisconsin, United States of America; 4 Department of Health Administration and Policy, College of Health and Human Services, George Mason University, Fairfax, Virginia, United States of America; University of Buea, Cameroon

## Abstract

**Objectives:**

Describe informal allopathic practitioner (IAP) knowledge and practice about management of hypertension and identify gaps in IAP knowledge and practice amenable to interventions.

**Methods:**

A cross sectional descriptive survey of 642 IAPs in Kamalapur (urban) and Mirsarai (rural) Bangladesh was conducted from March to April, 2011. Using a structured, pre-tested questionnaire sociodemographic, training, knowledge and practice data about management of hypertension was collected. Comparative statistics were preformed to show differences between urban and rural practitioners using SAS 8.0.

**Findings:**

99.4% of IAPs were male, mean age was 37.5 (12.5 SD) years. Greater than 65% correctly identified the upper limit of normal blood pressure. 50.2% underestimated lower limit of systolic hypertension. 79.8% allowed age to affect their treatment approach. As blood pressure increased, willingness to treat with medication decreased and tendency to refer increased. Sedative/sleeping pills, antidepressants, and beta blockers were the most commonly prescribed medications for prehypertension (58.7%, 50.3% and 53.7% respectively), stage I hypertension (55.0%, 38.6%, 49.8% respectively) and stage II hypertension (42.4%, 23.7%, and 28.8% respectively). Rural IAPs were more likely than urban IAPs to treat (84.7% vs 77.7%), order tests (27.1% vs 6.0%) and write prescriptions (60.4% vs 18.7%).

**Conclusion:**

While IAPs are crucial to Bangladesh’s pluralistic healthcare system, gaps in knowledge and practice could cause unnecessary harm. To include IAPs in the public sector’s fight against the chronic disease epidemic, interventions aimed at standardizing IAPs knowledge and practice will be essential. Successfully utilizing IAPs will have beneficial implications not only for Bangladesh, but for all developing countries.

## Introduction

The WHO recognizes primary care as a bed rock of cost effective, efficient health care. However, the developing world faces shortages of and lack of access to physicians [1]–[4]. This shortage of care providers contributes to the informal healthcare sector’s growing prominence in the field of primary care [5]–[9]. Informal allopathic providers (IAPs), comprised of village doctors and drug sellers, are particularly vital to health care in Bangladesh [5], [10], [11], providing as much as 65% of primary care [6]–[8].

A growing chronic disease epidemic is now making new demands of healthcare systems and providers. The steady increase of global chronic disease burden disproportionately affects low and middle income countries [12], [13]. In rural Matlab, Bangladesh, non-communicable disease mortality (excluding injury and accident) increased from 8% (1986) to 68% (2006) [14]. This epidemiological shift has serious implications for Bangladesh’s economy, healthcare system and society [9], [15]. Of the chronic diseases, hypertension is one of the world’s most prevalent [16], [17]. In more developed countries, hypertension screening was developed to catch this disease early and prevent serious complications through proper disease management. Unfortunately, routine hypertension screening is not as developed in Bangladesh, where prevalence estimates are as high as 18% [18], perhaps even higher. A significant healthcare workforce is required to confront this coming chronic disease epidemic.

A recent literature review concluded that despite importance of primary care health workers in developing countries, an inappropriate knowledge and practice persisted across the spectrum of training and specialty. The review further concluded that inadequate or nonexistent national guidelines frequently played a central role in these knowledge and practice deficits [19]. Despite the significant role of IAPs in Bangladesh’s healthcare network, IAPs have little contact with the government in terms of support, accountability or regulation [11], [20]. Studies on acute infectious disease internationally and in Bangladesh show IAPs often wrongly prescribe, diagnose and advise patients [11], [21], [22]. Some evidence has emerged indicating that IAPs play a significant role in hypertension management, but little is known about what is being done within that role [23]. While most Bangladeshi IAPs (71.5%) admit to treating hypertension patients, they also admit that diseases like hypertension are not prioritized in their training [21], [24].

IAPs are a primary source of healthcare in Bangladesh and could potentially be key actors in controlling the chronic disease epidemic. Before speculating at their potential role in the formal sector, data must be collected about their current knowledge and practice in the informal sector. This study aims to fill the gap in the literature by describing and comparing IAPs knowledge and practice in urban and rural Bangladesh.

## Methods

The study population was obtained from Health Demographic Surveillance System (HDSS) study populations at 2 sites; rural Mirsarai *upazilla* (a rural subdistrict in southeastern Bangladesh) and the urban Kamalapur surveillance site (an urban collection of 7 stratum in southeastern Dhaka). The total surveillance population in Mirsarai in 2009 was 39,025. Mirsarai’s average household income was 8,040 BDT per month (in 2008) median 6,000 BDT. Mirsarai’s disease profile is predominantly fever, digestive disturbance, and respiratory disease [25]. Total surveillance population in Kamalapur was 32,441. On average, one quarter of Kamalapur residents live below the poverty line, with a monthly income (in 2009) less than 13,902 taka per month. HDSS Kamalapur disease profile data is currently unavailable [26]. Both sites are associated with the International Center for Diarrheal Disease Research, Bangladesh (ICDDR,B) Health and Demographic Surveillance System (HDSS) and are part of the INDEPTH network. Ethical clearance for the study was obtained from the ICDDR,B review board.

Cross sectional data was collected on IAPs from March to April 2011 by trained ICCDDR,B field research assistants and field research officers based out of ICDDR,B field research offices in the respective surveillance sites. ICDDR,B field offices are long established satellites of the ICDDR,B used for data collection involving ICDDR,B research projects and population surveillance activities. The study population was limited to persons over 18 years old practicing allopathic medicine within the study sites. Persons practicing mixed medicine (allopathic medicine plus ayurvedic, traditional, or herbal medicines) or allopaths with MBBS degrees were excluded. Eligible respondents claimed to hold varying levels of certifications ranging from none at all or a simple Drug License obtained in a few months, a 3–6 month Local Medical Assistant and Family Planning training (LMAF) degree, a 3–6 month Primary Medical Counselor (PMC) degree, a 3 year Diploma in Medical Faculty (DMF), a 1 year Rural Medical Practitioner (RMP) degree. However, holding a given certificate does not always reflect the quality of education or the implied length of training as there is little oversight of training institutions. [21] Preexisting lists of eligible respondents developed by ICDDR,B field site satellite offices were cross checked by field researchers who interviewed respondents for names and locations of nearby IAP businesses. Names were cross checked with existing lists to create a comprehensive list, identifying all eligible respondents within the predefined study area. Trained field research assistants conducted interviews at the IAP’s place of business during work hours. Unavailable practitioners were contacted by mobile phone to schedule interviews.

A total of 733 possible respondents were approached, 731 gave informed consent for the interview, 89 were ineligible (MBBS degree, practiced mixed therapy etc…) for inclusion in this study. Final sample size was 642 respondents (391 in Mirsarai, 251 in Kamalapur). The original sample size target was 390, to ensure adequately powered descriptive analysis. This target was expanded to achieve sufficient power for urban rural comparisons.

A structured survey was designed to collect descriptive data on IAP hypertension knowledge and practice (See Supporting Information). Survey questions and categorical responses were based on previous studies, hypertension practice guidelines, IAP interviews and focus groups discussions (FGDs) [21], [27], [28]. FGDs covered most key issues categorized in the questionnaire (i.e. appropriate hypertension management, cause of hypertension, symptoms of hypertension ect…). The questionnaire was developed in English, translated to Bangla and then back translated to English, before being pretested outside the study areas in urban (Mirpur) and rural (Matlab) Bangladesh.

Surveys conducted in Kamalapur were designated urban while those conducted in Mirsarai *upazilla* were designated rural. Distinctions were made between urban and rural IAPs based on previous literature suggesting possible differences in practice between these two groups [20], [29]. Responses were evaluated according to international (JNC and WHO) guidelines; in place of national guidelines, a training module/guidelines developed by the WHO and the national government of Bangladesh were also included in evaluations. Hypertension was classified as stage I hypertension (140/90–159/99 mmHg) and stage II hypertension (>160/100 mmHg) [30]. Respondents were asked about diagnosis (identifying a new cases of hypertension in their role as IAP), prescription (recommending a medication, dosage and plan on paper), advice (verbally advising the patient on a course of action regarding their disease), and referral (instructing the patient to seek care from another practitioner).

Descriptive analyses were performed on demographic characteristics, professional training, knowledge, treatment, prescribing, and referral practices. Urban and rural differences were evaluated with chi-square tests and independent sample t-tests where appropriate. Multivariate analysis was preformed looking at age religion, experience and training that affected the likelihood of appropriately defining hypertension. SAS 8.0 was used to perform all statistical analysis.

## Results

IAPs were 99.4% male with a mean age of 37.5 (12.5 SD) years. 61.3% earned more than 7,500 taka, US$ 107, (1 USD: 70 BDT) per month, however, 47.2% reported being in debt. 60.8% reported education beyond the secondary level (greater than 10 years), urban IAPs had more education and higher incomes than rural IAPs (Table 1).

**Table 1:** IAP Sociodemographic Variables.

Variables	All	Urban	Rural	p-value
Total Sample (n)	642	251	391	
Gender (Male)	99.4	99.2	99.5	0.6461
Age Mean (SD)	37.5(12.5)	35.3(11.6)	38.9(12.9)	0.0003
**Education**				
1–5 years6–10 years11–12 years>12 years	0.938.339.920.9	1.227.943.427.5	0.845.037.616.6	<0.0001
**Supplementary Occupation**				
NoneAgricultureServiceOther	91.72.03.74.2	94.00.02.05.2	90.33.34.93.6	0.1060.0020.0860.323
**Average Monthly Income (in BDT.)**				
<2,5002,500–5,0005,001–7,5007,501–10,000>10,000	2.518.417.827.733.6	0.412.416.331.139.8	3.822.318.725.629.7	<0.0001
**Poverty Status**				
Always deficitBreak-evenSurplus	47.239.113.7	44.241.014.7	49.137.913.0	0.2309
**Religion**				
MuslimHinduBuddhismOther	62.936.00.90.2	82.516.70.40.4	50.448.31.30.0	<0.0001
**Marital Status**				
MarriedUnmarried	71.328.7	72.127.9	70.829.2	0.789

SD-Standard Deviation, BDT-Bangladesh Taka.

**Table 2:** IAP Training.

Variables	All	Urban	Rural	p-value
**Entry into profession**				
Formal TrainingInformal TrainingSelling MedicineServiceInherited*	58.67.322.45.16.5	51.46.028.35.29.2	63.28.218.75.14.9	0.004
**Certification**				
Drug LicenseLMAFPMCDMFRMPOtherNone	22.341.034.610.416.01.317.9	19.542.640.68.010.00.420.3	24.039.930.712.020.01.816.4	0.2060.5110.0110.1130.00040.1580.207
**Training Institution**				
Thana(UZ) health complexThana level privateDistrict hospitalDistrict level privateNGOOther (Description)	27.48.012.766.46.02.0	9.913.013.073.35.61.2	37.25.212.562.56.32.4	<0.00010.0060.8830.0220.7780.500
**Topics Covered in Training**				
Diarrheal diseaseFever/Common Cold CoughDiabetesAsthmaHeart DiseaseHypertensionOther (Description)	98.799.132.163.935.990.44.2	100.0100.024.2 49.128.087.61.9	97.998.636.572.240.392.05.7	0.0930.3020.008<0.00010.0100.1350.085
**Equipment**				
StethescopeSphygmanometer	97.797.7	100.099.6	96.296.4	0.0010.007
**Location of Service Provision**				
Drug ShopVillageHealth facilityHouse callsOther (description)	99.536.61.465.90.2	99.68.00.069.30.0	99.555.02.363.90.3	1.000<0.00010.0140.1481.000

IAP- Informal Allopathic Provider, LMAF- Local Medical Assistant and Family Planning training, PMC-Primary Medical Counsel, Diploma in Medical Faculty, Rural Medicine Practitioner, UZ-Upazilla, BRAC-Bangladesh Rural Advancement Committee, ICDDR,B International Center for Diarrheal Disease Research, NGO-Non-Governmental Organization.

* Inherited entry- when an individual became an IAP through a relative already in the profession.

** all persons having received training were included; those stating no training were excluded.

The majority of IAPs entered their profession through formal training from a private organization (58.6%) (rural 51.4% vs urban 63.2%). 17.9% of IAPs had no training certification. The most common certifications were LMAF and PMC. 99.5% of IAPs practiced from a drug shop, 65.9% also made house calls. 97.7% possessed equipment for measuring blood pressure (sphygmanometer and stethoscope). 90.4% self reported receiving hypertension training (Table 2).

**Table 3:** IAP Hypertension Knowledge.

Variables	All	Urban	Rural	p-value
**Patient age affect your approach to Hypertension?** (1 = yes)	79.8	77.7	81.1	0.3150
**Normal Blood Pressure (upper limit)**				
Age <60				
Systolic <120 Systolic 120 Systolic >120* Systolic Mean (sd) mmHg* Diastolic <80 Diastolic 80 Diastolic >80* Diastolic Mean (sd) mmHg*	5.165.329.6*124(11)*9.267.323.5*82(8)*	3.265.331.5*124(12)*2.869.327.9*83(9)*	6.465.228.4*124(12)*13.366.020.7*80(8)*	0.170*0.680*<0.0001*<0.0001*
Age >60				
* Systolic Mean (sd) mmHg* * Diastolic Mean (sd) mmHg*	*138(15)* *90(11)*	*136(12)* *91(11)*	*138(17)* *89(11)*	*0.041* *0.002*
**Hypertension (lower limit)**				
Age <60				
Systolic <140 Systolic 140 Systolic >140* Systolic Mean (sd) mmHg* Diastolic <90 Diastolic 90 Diastolic >90* Diastolic Mean (sd) mmHg*	50.237.112.8*137(12)*25.957.316.8*89(7)*	51.439.49.2*136(8)*16.766.117.1*90(6)*	49.435.515.1*137(14)*31.751.716.6*88(7)*	0.084*0.057*<0.0001*<0.001*
Age >60				
* Systolic Mean (sd) mmHg* * Disastolic Mean (sd) mmHg*	*148(16)* *96(10)*	*147(11)* *98(9)*	*149(18)* *96(10)*	*0.035* *0.1462*
**Most common causes of hypertension?**				
Stress Genetic Inheritance High Cholesterol Poor Diet Eating Too Much Salt Lack of Sleep Irregular routine Tobacco Products Alcohol Other (Description)	61.416.839.98.322.624.87.06.78.10.2	64.114.735.911.621.926.79.68.811.20.0	59.618.242.56.123.023.55.45.46.10.3	0.2800.2810.0990.0180.7720.3990.0560.1060.0261.000

IAP- Informal Allopathic Provider, HTN-Hypertension, SD-Standard Deviation, mmHg-millimeters of Mercury.

82.7% of IAPs defined hypertension as elevated blood pressure with stress being the most common cause (61.4%). Only 22.6% of IAPs ranked salt consumption among the top 3 most common causes. 79.8% acknowledged that patient age affects their practice. When asked to identify the upper limit of normal blood pressure in patients below the age of 60, 65.3% correctly identified a systolic pressure of 120 mmHg and 67.3% correctly identified a diastolic pressure of 80 mmHg (mean 82 mmHg SD 8). For patients over the age of 60, the majority of IAPs overestimated the upper limit of normal blood pressure for systolic (120 mmHg) and diastolic (80 mmHg). For patients under the age of 60, 50.2% of IAPs underestimated the lower limit of systolic hypertension as less than 140 mmHg (mean 137 mmHg SD 12); 57.3% correctly defined the lower limit of diastolic hypertension at 90 mmHg (mean 89 mmHg SD 7). For patients over the age of 60, the majority of IAPs overestimated the lower limit of systolic (140 mmHg) and diastolic (90 mmHg) hypertension (Table 3).

**Figure 1:**
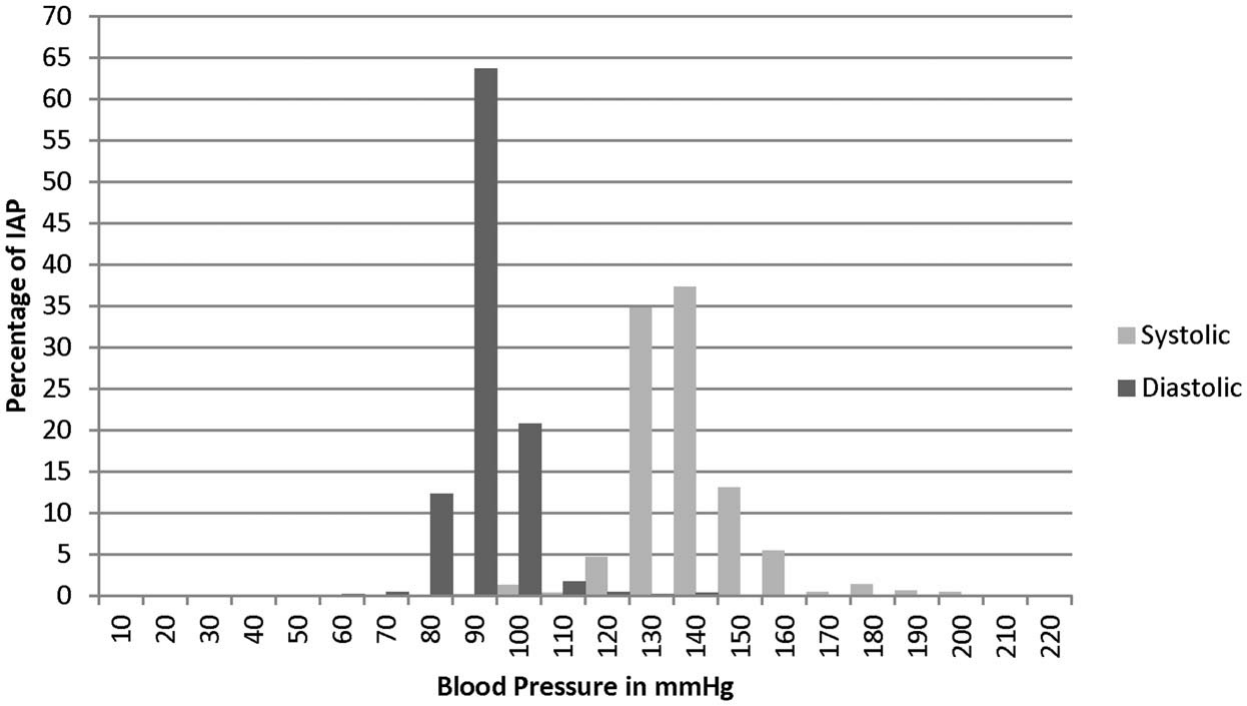
Lower Limit of Blood Pressure at which to initiate medication in <60 YO*. IAP- Informal Allopathic Practitioner, mmHg- millimeters of mercury. *Medication should typically be initiated at 140/90 mmHg.

**Table 4:** IAP Hypertension Treatment Practices.

Variables	All	Urban	Rural	p-value
**HTN Patient Services**				
AdviceTreatmentRefer to specialistWrite perscriptions	100.081.996.144.1	100.077.793.618.7	100.084.797.760.4	0.0280.012<0.0001
**Lower limit of HTN for intiating medication**				
Age <60				
* Systolic Mean (sd)mmHg* * Diastolic Mean (sd)mmHg*	139(13)91(7)	138(12)92(8)	139(14)91(7)	0.0970.010
Age >60				
* Systolic Mean (sd) mmHg* * Diastolic Mean (sd) mmHg*	151(15)97 (11)	148(12)99(12)	153(16)96 (10)	<.00010.002
**Treatment Duration**				
<12 months1–2 Years>2 yearsForeverUntil free of symptomsUntil BP is normal	0.20.20.347.413.638.5	0.00.40.442.614.741.8	0.30.00.350.412.836.3	0.318
**Do you refer?**				
NeverRarelySometimesOftenAlway	0.51.348.429.820.1	0.41.630.342.625.1	0.51.060.121.516.9	<0.0001
**Most common referral site**				
MBBSMBBS SpecialistCardiologistGovernment HospitalVillage DoctorDiagnostic CenterNational Heart Foundation	26.630.17.930.42.70.81.6	26.739.85.623.10.00.34.0	26.623.89.535.04.40.80.0	<0.0001
**Blood Pressure at which referal is necessary**				
Age <60				
* Systolic Mean (sd)mmHg* * Diastolic Mean (sd)mmHg*	163(21)102(12)	161(19)103(13)	164(23)101(12)	.100.036
Age >60				
* Systolic Mean (sd) mmHg* * Diastolic Mean (sd) mmHg*	175(23)108(14)	169(21)108(14)	179(24)109(14)	<.0001.489

IAP- Informal Allopathic Provider, HTN-Hypertension, MBBS-Medical Bachelors and Bachelors of Surgery, SD-Standard Deviation, mmHg-millimeters of Mercury, BP-Blood Pressure.

**Table 5:** IAP Medication Practices*.

Variables	All	Urban	Rural	p-value
**Anti depressant**				
120/80 to 140/90140/90–160/100160/100+	50.338.623.7	47.026.315.5	52.446.628.9	0.196<0.0001<0.0001
**Sedative/Sleeping Pill**				
120/80 to 140/90140/90–160/100160/100+	58.755.042.2	57.851.428.7	59.357.350.0	0.7430.145<0.0001
**Beta Blocker**				
120/80 to 140/90140/90–160/100160/100+	53.749.828.8	57.845.424.7	51.252.731.5	0.1050.0760.074
**CCB**				
120/80 to 140/90140/90–160/100160/100+	30.846.731.5	27.542.223.9	33.049.636.3	0.1610.075<0.0001
**Diuretic**				
120/80 to 140/90140/90–160/100160/100+	5.913.615.7	2.43.66.0	8.220.022.0	0.002<0.0001<0.0001
**Losartan Potassium**				
120/80 to 140/90140/90–160/100160/100+	15.036.141.0	8.819.920.3	18.946.654.2	<0.001<0.0001<0.0001
**ACE Inhibitor**				
120/80 to 140/90140/90–160/100160/100+	2.04.76.7	3.25.65.6	1.34.17.4	0.1480.4440.420
**Other (description)**				
120/80 to 140/90140/90–160/100160/100+	0.61.12.3	0.40.40.4	0.81.53.6	1.0000.2560.007
**None**				
120/80 to 140/90140/90–160/100160/100+	22.625.144.2	23.537.165.0	22.017.431.0	0.699<0.0001<0.0001

CCB-Calcium Channel Blocker.

* Commonly prescribed medications were given by brand and generic name under each class of drug as follwos: Anti depressant Frengit or Meltix/Flupentixol Meletracin, Sedative/Sleeping Pill Bupam/Bomazepam; Clobazam/Frizium Beta Blocker Teneloc/Atenolol; Indever/Propanolol CCB Amdocol/Amlodipine; Nipin/Nefidipine, Diuretic Lasix/Furosemide; Dezide/HZT, Losartan Potassium Osartil, ACE inhibitor Altace or Piramil/Ramipril.

100% of IAPs reported giving hypertension patients advice while only 81.9% of IAPs reported treating hypertension patients. Compared to urban IAPs, rural IAPs were more likely to treat (77.7% vs 84.7%), order tests (6.0% vs 27.1%) and write prescriptions (18.7% vs 60.4%) (table 4). As blood pressure increased, IAPs willingness to attempt treatment decreased. Urban IAPs, compared to rural IAPs, were more likely not to treat patients at stage I hypertension (140/90–159/99 mmHg) (37.1% vs 17.4%) and stage II hypertension (≥160/100 mmHg) (65.0% vs 31.0%). Compared to urban IAPs, rural IAPs prescribed medication with greater frequency, with the exception of ACE inhibitors and beta blockers for prehypertensives as well as ACE inhibitors for stage I hypertension. Sedative/sleeping pills, antidepressants, and beta blockers were the most commonly prescribed medications for prehypertension (58.7%, 50.3% and 53.7% respectively), stage I (55.0%, 38.6%, 49.8% respectively) and stage II (42.4%, 23.7%, and 28.8% respectively) (Table 5). In patients under the age of 60, 34.8% and 37.3% of IAPs advocated starting medication at a systolic pressure of 130 and 140 mmHg respectively; 63.7% advocated starting medication at 90 mmHg diastolic (Figure 1). In patients over the age of 60, the blood pressure at which medications were initiated was higher than in patients less than 60. IAPs most commonly advocated medicating hypertension indefinitely (47.4%), the next most common response (38.5%) was to medicate until blood pressure returned to normal (Table 4).

**Figure 2:**
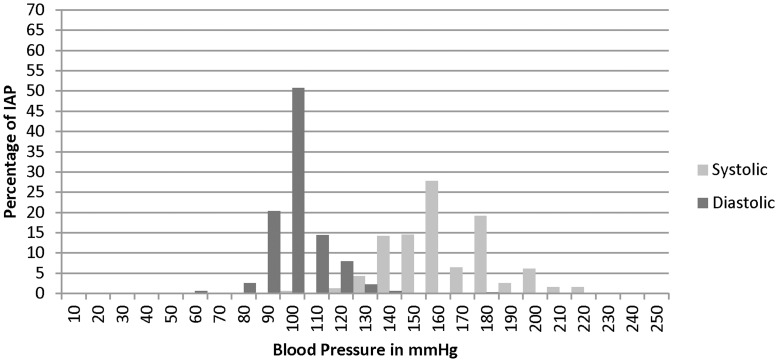
Blood Pressure at which to refer patients <60 YO*. IAP- Informal Allopathic Practitioner, mmHg- millimeters of mercury. *There is no recommended blood pressure for referral among international guidelines. BP>160/100 is considered stage III HTN and should likely be referred for more skilled management.

96.1% of IAPs reported “making referrals” as part of their care for hypertensive patients. 49.9% reported referring patients often or always, while 48.4% reported referring patients sometimes. Urban IAPs indicated a higher frequency of referring often or always compared to rural IAPs (67.7% vs 38.4%). 56.7% of referrals were made to a specific MBBS/MBBS specialist, while 30.4% were to government hospitals (Table 4). Most IAP referrals occurred at or below 160/100 mmHg. For those under the age of 60, IAPs commonly advocated making referrals at a systolic pressure of 160 mmHg (27.8%), for diastolic 100 mmHg (50.7%) (Figure 2). For those over the age of 60, IAPs advocated referring at a higher systolic pressure than those less than 60 (Table 4).

Multivariable analysis was preformed to account for the effect of confounders and covariates on proper identification of hypertension by IAP. No statistically significant results were identified using multivariate analysis except for a decreased probability (OR 0.48) that IAPs holding drug licenses could correctly identify hypertension in patients less than 60 years old compared to IAPs with other certifications (Table 6).

**Table 6:** Multivariate Analysis of Potential Factors Influencing Proper Identification of Hypertension by IAP.

	≤60 Years Old			> 60 Years Old		
	OR	95% CI		OR	95% CI	
Age	1.00	0.98	1.02	0.99	0.97	1.02
**Formal Education**						
1 to 5 years	Ref			Ref		
6 to 10 years	1.40	0.16	12.61	0.22	0.04	1.15
11 to 12 years	1.74	0.19	15.65	0.25	0.05	1.33
13 years or more	1.68	0.18	15.44	0.24	0.04	1.33
**Religion**						
Muslim	0.97	0.18	5.24	1.26	0.14	11.18
Hindu	0.81	0.15	4.44	1.56	0.17	13.94
**Professional Experience**						
≤5 years	Ref			Ref		
6 to 14 years	1.25	0.76	2.06	1.38	0.78	2.43
15 years or more	1.89	0.99	3.62	1.82	0.85	3.90
**Training Certification**						
Drug License	**0.48**	**0.29**	**0.79**	1.26	0.73	2.15
LMFP	1.03	0.99	3.62	0.95	0.60	1.48
RMP	1.13	0.29	0.79	0.68	0.34	1.36
**Training Received**						
Any Training	1.08	0.36	2.52	0.43	0.11	1.65
Formally Trained	0.66	0.39	1.16	0.58	0.31	1.10
Hypertension Training	1.77	0.79	4.01	2.54	0.75	8.56
**Equipment**						
Owns Sphygmanometer	1.74	0.37	8.20	3.23	0.40	25.86
**Location**						
Urban Site	1.02	0.68	1.54	1.04	0.64	1.67

IAP- Informal Allopathic Providers, OR- Odds Ratio, CI- Confidence Interval, Ref- Reference Variable, LMAF- Local Medical Assistant and Family Planning training, RMP- Rural Medical Practitioner.

## Discussion

### Knowledge Gaps

Many gaps in IAPs knowledge (i.e. when to diagnose, medicate, discontinue medication and what medications to prescribe) interfered with proper hypertension management, particularly in patients over age 60. Bangladeshi and international treatment guidelines do not adjust their recommendations based on age [27], [28], [31]. Lack of knowledge among IAPs regarding appropriate blood pressure goals leads to mismanagement of hypertensives and non hypertensives alike. The recommended treatment, diuretics (i.e. thiazide) [32], was under prescribed while inappropriate treatments (sedatives and antidepressants) [33] were over prescribed. Under treatment of hypertension and inappropriate prescribing practices increase the risk of cardio and cerebrovascular events.

There are several potential explanations for these knowledge and practice gaps. Previous studies suggest that low standards of education required for certification and lack of continuing education are responsible for substandard IAPs practices [11], [21]. Existing sources of continuing education, like pharmaceutical representatives, may also be biased and misinformed [20], [33]. Furthermore, IAPs in other countries are documented as mimicking physician prescribing practices. When patients fill the MBBS doctor’s prescription at an IAP’s drug shop, IAPs will take note or interview that patient about their symptoms. When a patient with similar symptoms comes to the shop before being seen by an MBBS, the IAPs will mimic the MBBS doctor by prescribing that drug [34]. Such behavior could implicate Bangladeshi MBBS as a source of misinformation. Access to quality information sources are limited and likely underlie IAPs hypertension knowledge and practice gaps.

### Risk Adverse Behavior

Early referral and reluctance to treat patients with higher blood pressures points towards “adverse to risk” behavior among IAPs. Despite direct financial incentive to medicate [24], few IAPs prescribed for patients with higher blood pressures while referrals and advice were frequent even at pre hypertension and stage 1 hypertension. A qualitative study by Ashraf et al. 1982 suggested that IAPs were not held accountable for malpractice secondary to “fatalistic attitudes” of villagers [35]. Although cited in recent literature this study was written almost 30 years ago, since that time the professional landscape of the IAPs has changed. Market forces, as opposed to education, are likely to strongly influence IAPs to be adverse to risk with their practice. Complicated patients are more likely to deteriorate, even die; for IAP, being identified as the primary provider of a complicated patient is risky, even bad for business. As blood pressure increases, IAPs seem reluctant to adopt a prescribing role after identifying hypertension, opting to give patients advice and refer them to MBBS or specialists. IAPs are more likely to live in the village or a nearby town and may be better suited to give advice and explain concepts to the poor and uneducated as opposed to an educated doctor with an overburdened practice from a different socioeconomic class. That being said it will be crucial to make sure that the advice being given is appropriate and well informed. It may be possible to expand on current disease screening practices and IAPs’ willingness to dispense advice instead of drugs creating potential roles for IAPs in a more formal system.

### Urban vs Rural

Urban and rural IAPs were distinguished by important sociodemographic variables (income, religion and training) as well as practices. Overall IAPs stopped attempting treatment earlier than expected, but compared to more educated and higher paid urban IAPs, rural IAPs prescribed more medications and waited to refer patients until higher blood pressures were reached. Given their lower incomes they are at particular risk of being financially influenced in their decision making practices. Rural IAPs have financial incentive to delay referrals to MBBS, but if financial incentives were the primary driving factor, they would also be expected to prescribe medications at lower blood pressure goals than urban IAP, which they do not. Unfilled rural doctor postings, and high rates of absenteeism make MBBS a less viable health care option for many rural Bangladeshis [20], [29]. Transportation expenses, logistics and hidden service costs further compound the MBBS care seeking scenario [11], [36]. Rural IAPs may be more willing to attempt treatment because they recognize the limited options available to their patients. Rural IAPs are particularly crucial in health care coverage and despite poor knowledge and practice, are often the only option for poor rural Bangladeshis. These urban rural differences between IAPs are a particularly important consideration for policy makers and those considering a partnership between IAPs and the formal sector.

### Study Strengths

There are several strengths to our study. First our study had a relatively large sample (639 participants), making it one of the larger studies of IAPs to date in Bangladesh and internationally. Our sample compared urban and rural IAP practices; a comparison not seen in previous studies, despite a growing body of literature indicating serious differences in urban versus rural health care [20], [37]. The more narrow operational definition of IAPs allowed the study to focus on potential candidates for partnerships with the formal sector. Our study enjoyed nearly 100% participation from the IAPs community mirroring similarly high levels of compliance in previous IAPs studies [21]. The high rate of participation helps to ensure the accuracy of our findings.

### Study Limitations

There are some important limitations to this study. While not nationally representative, our study was all inclusive of IAPs in the study area and baseline characteristics reflected those in previous studies. Although self reported data has long been used as an epidemiological tool, reporting bias creates a risk of intentional or accidental omissions of actual IAP practices. Therefore the information presented in this study is a theoretical description of IAPs knowledge of “best practice” for hypertension management. Direct observation of IAPs or testing with simulated clients might capture IAP knowledge and practice more accurately, but neglects informed consent and requires the logistical and technical difficulties of imitating hypertension symptoms. Selection bias for even numbers ending in zero is a likely explanation of atypical distribution patterns in our blood pressure figures. Data was not collected on IAPs therapeutic goals, although treatment duration data provides some insights.

The base line characteristics of the study population were similar in most respects to those in previous Bangladeshi IAP studies [21], [24], [38]. Adjusting for annual inflation rates accounted for differences in IAP incomes [39]. Our study sample reported more years of education than seen in previous studies [21], [38]. This is likely due to variations among the Bangladeshi population dependent on geography and time. However, reporting bias in which respondents exaggerate their level of education must also be considered.

### Contextualization of Results

Significant gaps in IAP hypertension knowledge were an expected outcome of the study based on previous IAP and MBBS knowledge and practice studies in Bangladesh and elsewhere (13,25–30). Knowledge and practice gaps in our study were found among MBBS in developing and developed countries (33,40–44) Comparison with similar international studies among trained physicians indicate that while MBBS are less likely to have gaps in knowledge and practice, their knowledge and practice deficits are likely to be similar.

### Next Step

The likely harm caused by current IAP knowledge and practice casts doubt on the efficacy or wisdom of immediately utilizing IAP as formal sector partners in treating hypertension. However, the adverse to risk behavior and capability to detect hypertension among poor rural Bangladeshis suggest that it may be appropriate to further encourage and formalize their attempts to screen patients for hypertension. Encouraging such practices offers a first step toward harm reduction and strengthening linkages with the formal sector. IAPs participating in a screening program with the formal sector may then be further evaluated, allowing for potential expansion of their role combating the hypertension (or chronic disease) epidemic. Further research assessing IAP skill in identifying health complications related to hypertension should pay particular attention to speed of referral to appropriate care and recognition of severity of complications.

Control of chronic disease in low and middle income countries is a global issue one that relies on a primary care approach emphasizing team based care and formal linkages to communities [40]. IAPs are the dominant primary care providers in rural areas and will continue to encounter hypertension patients. Interventions are needed to improve knowledge and practice as well as reduce harm. Approaches to improve hypertension knowledge and practice among PCP in other countries may be applicable to IAPs in Bangladesh. IAPs in Bangladesh and elsewhere have already shown high rates of participation in interventions aimed at improving their knowledge and practice [21], [22], [40]–[44].

### Conclusion

Bangladeshi IAPs have identifiable and possibly deleterious gaps in their hypertension knowledge and treatment behaviors. Because IAPs inhabit a particularly important role within the Bangladesh health system IAPs nor the aforementioned gaps can be ignored. By describing IAPs hypertension knowledge and practice, future research can study the efficacy of interventions aimed at changing IAPs practice and patient outcomes. To include IAPs in the public sector’s fight against the chronic disease epidemic, interventions aimed at standardizing IAPs knowledge and practice will be essential. However, policy makers might choose to consider IAPs limitations before officially incorporating them into the health care delivery system. Successfully utilizing IAPs will require that their limitations be fully addressed so that the beneficial implications of their collaboration can be appreciated not only in Bangladesh, but for all developing countries.

## Supporting Information

Questionnaire S1 (DOC)
