# Supplementary material for: Informal Allopathic Provider Knowledge and Practice Regarding Hypertension in Urban and Rural Bangladesh
Source: PLoS One. 2012 Oct 25;7(10):e48056. doi: 10.1371/journal.pone.0048056 (PMC3485017; doi:10.1371/journal.pone.0048056)
Supplement: Questionnaire S1 — (DOC) [file pone.0048056.s001.doc]

**International Centre for Diarrhoeal Disease Research, Bangladesh (ICDDR,B)**

Protocol Number PR-10065

**Protocol title:** Chronic disease knowledge attitude and practice among unqualified allopathic practitioners in urban and rural Bangladesh.

Ques. Serial No:

| Date of interview:    Day Month Year | | | | |
| --- | --- | --- | --- | --- |
| Interviewers name and code: **------------------------------------------------------------** | | |  | |
| Interview result:  Completed Interview ­ 1  Incomplete, reason 2  Refusals, reason 3 | | |  | |
| Supervisors Signature: ------------------------------------------------------Date: ---------/--------/2011  Instruction to the Interviewer: If you want to write anything descriptive please write it in the space provided under the question | | | | |
| Social and demographic data | | | | |
| No | Question and filter | Coding categories | | **SKIP** |
| **A.** | Record the starting time | Minute Hour | |  |
| 101 | Gender | Male 1  Female 2 | |  |
| 102 | How old are you? (In completed years) |  | |  |
| 103 | How many years of formal schooling and or college have you completed? | None 1  1-5 2  6-12 3  11-12 4  ≥13 5 | |  |
| 104 | What is your religion? | Muslim 1  Hindu 2  Buddhism 3  Christianity 4  Other (description) 5 | |  |
| 105 | What is your current marital status? | Married 1  Unmarried 2  Divorced/separated 3  Widowed 4 | |  |
|  |  |  | |  |
| 106 | Do you have a supplementary occupation?  Yes------1  No------2 | None  Agriculture  Service  Other (description) | |  |
| 107 | What is your monthly household income (in a normal month)? (in Takas) | ≤2500 1  2501-5000 2  5001-7500 3  7501-10,000 4  >10,000 5 | |  |
| 108 | How would you describe your financial situation? | Always Deficit 1  Break-even 2  Surplus 3 | |  |
| 109 | What do you consider yourself to be | Village Doctor 1  Drug Seller 2  Both 3  Other (Description) 4 | |  |
| 110 | How long have you been a village doctor/ drug seller (In completed years)? |  | |  |
| 111 | How did you become a village doctor/drug seller? | Formal Training 1  Informal training apprenticeship 2  Selling Medicine 3  Service 4  Hereditary 5  Other (description) 6 | |  |
| 112 | What are your currently valid training certificates?  Yes------1  No------2 | 1. Drug License 2. LMAF 3. PMC 4. DMF 5. RMP 6. MBBS 7. Other (description) 8. Other (description) 9. None | |  |
| 113 | Have you ever received training in providing healthcare? (Yes=1 No=2) | Yes 1  No 2 | | **If No, go to Q118** |
|  |  |  | |  |
| 114 | What topics were covered during your training?  Yes------1  No------2 | 1. Diarrheal disease ------- 2. Fever/Common Cold Cough 3. Anatomy 4. Skin disease 5. Digestive disturbances 6. Family planning 7. Anemia 8. Tuberculosis 9. Physiology 10. Chronic Disease 11. Cancer 12. Diabetes 13. Asthma 14. Heart Disease 15. Hypertension 16. Other (Description) | |  |
| 115 | Duration of Training Completed (in Months) |  | |  |
| 116 | Was this training sufficient for the services the villagers request you to perform? | Yes 1  No 2 | |  |
| 117 | From where did you receive your training?  Yes------1  No------2 | 1. Thana(UZ) health complex 2. District hospital 3. District level private organization 4. BRAC 5. ICDDR,B 6. Other NGO 7. Thana level private organization 8. Other (Description) | |  |
| 118 | Do you have a blood pressure measuring machine? | Yes 1  No 2 | |  |
| 119 | Do you have a Stethoscope | Yes 1  No 2 | |  |
| 120 | At which of the following places do you provide services?  Yes------1  No------2 | 1. Drug Shop 2. Village 3. Health facility 4. House calls 5. Other (description) | |  |
| 121 | Do you prescribe or practice something besides allopathic medicine?  Yes------1  No------2 | 1. Traditional 2. Ayurvedic 3. Homeopahic 4. Spiritual 5. Herbal 6. Other (description) | |  |
| 122 | Which of the following best defines hypertension?  Description: | High Blood Pressure 1  Too Much Tension/Stress 2  Other (description) 3 | |  |
| 123 | Does patient age affect how you define hypertension? | Yes 1  No 2 | |  |
| 124 | In an otherwise healthy adult, what is the highest reading on your blood pressure machine that you are willing to say is normal blood pressure?  If the patient is 18 to 60 years old?  If the patient is more than 60 years old?  1) Systolic  2)Diastolic | 1) 2)  <60 a) /    >60 b) / | |  |
| 125 | In an otherwise healthy adult, at what reading on your blood pressure machine do you decide someone has hypertension?  If the patient is 18 to 60 years old?  If the patient is more than 60 years old?  1) Systolic  2)Diastolic | 1) 2)  <60 a) /    >60 b) / | |  |
|  |  |  | |  |
| 126 | What are the main causes of hypertension in Bangladesh?  Yes------1  No------2 | 1. Stress 2. Family Problems 3. Financial Problems 4. Heart Problems 5. Kidney Problems 6. Genetic Inheritance 7. High Cholesterol 8. Poor Diet 9. Easting Too Much 10. Eating Too Much Beef 11. Eating Too Much Salt 12. Lack of Sleep 13. Irregular routine 14. Tobacco Products 15. Alcohol 16. Other (Description) | |  |
| 127 | What are the top three most common causes of hypertension in Bangladesh?  Yes------1  No------2  [List only 3 responses] | 1. Stress 2. Family Problems 3. Financial Problems 4. Heart Problems 5. Kidney Problems 6. Genetic Inheritance 7. High Cholesterol 8. Poor Diet 9. Easting Too Much 10. Eating Too Much Beef 11. Eating Too Much Salt 12. Lack of Sleep 13. Irregular routine 14. Tobacco Products 15. Alcohol 16. Other (Description) | |  |
|  |  |  | |  |
| 128 | What are the most common presentations of hypertension in your experience?  Yes------1  No------2 | 1. Patient Requests a BP check---------- 2. Headache--------------------------------- 3. Neck pain--------------------------------- 4. Chest pain-------------------------------- 5. Vertigo------------------------------------ 6. Lack of sleep----------------------------- 7. Urinary problems----------------------- 8. Anxiety----------------------------------- 9. Depression------------------------------- 10. Referral from Drug seller-------------- 11. Not feeling well----------------------- 12. Falling down----------------=----------- 13. Nausea----------------------------------- 14. Other **(**Description**)**--------------------- | |  |
| 129 | What are the top 3 most common presentations of hypertension in your experience?  Yes------1  No------2  [List only 3 responses] | 1. Patient Requests a BP check---------- 2. Headache--------------------------------- 3. Neck pain--------------------------------- 4. Chest pain-------------------------------- 5. Vertigo------------------------------------ 6. Lack of sleep----------------------------- 7. Urinary problems----------------------- 8. Anxiety----------------------------------- 9. Depression------------------------------- 10. Referral from Drug seller-------------- 11. Not feeling well----------------------- 12. Falling down----------------=----------- 13. Nausea----------------------------------- 14. Other **(**Description**)**--------------------- | |  |
| 130 | How many times do you measure blood pressure before you are confident that the patient has hypertension? |  | |  |
| 131 | Over how many days do you measure blood pressure to get an accurate reading? |  | |  |
|  |  |  | |  |
| 132 | Which services do you offer your patients with hypertension?  Yes------1  No------2 | 1. Advice 2. Treatment 3. Refer to specialist 4. Order tests 5. Write perscriptions 6. Keeps patient records 7. Other (Description) | |  |
| 133 | What advice do you give someone with hypertension?  Yes------1  No------2 | 1. Monitor Blood Pressure 2. Take Medication Regularly 3. Visit an MBBS Doctor 4. Change/Control Diet 5. Avoid Stress 6. Change Habits/(tobbacco) 7. Other (Description) | |  |
| 134 | At what blood pressure should one treat an otherwise healthy adult with hypertension medication ?  If the patient is 18 to 60 years old?  If the patient is more than 60 years old?  1) Systolic  2) Diastolic | 1) 2)  <60 a) /    >60 b) / | |  |
| 135 | What medication should one give someone with blood pressure of 120/80-140/90?  Yes------1  No------2 | 1. Anti depressant   (Frengit or Meltix/Flupentixol Meletracin)   1. Sedative/Sleeping Pill   (Bupam/Bomazepam; Clobazam/Frizium)   1. Beta Blocker   (Teneloc/Atenolol; Indever/Propanolol)   1. Calcium channel blocker   (Amdocol/Amlodipine; Nipin/Nefidipine   1. Diuretic   (Lasix/Furosemide; Dezide/HZT)   1. Losartan Potassium   (Osartil)   1. ACE inhibitor   (Altace or Piramil/Ramipril)   1. Other (description) 2. None | |  |
|  |  |  | |  |
| 136 | What medication should one give someone with blood pressure of 140/90-160/100  Yes------1  No------2 | 1. Anti depressant   (Frengit or Meltix/Flupentixol Meletracin)   1. Sedative/Sleeping Pill   (Bupam/Bomazepam; Clobazam/Frizium)   1. Beta Blocker   (Teneloc/Atenolol; Indever/Propanolol)   1. Calcium channel blocker   (Amdocol/Amlodipine; Nipin/Nefidipine   1. Diuretic   (Lasix/Furosemide; Dezide/HZT)   1. Losartan Potassium   (Osartil)   1. ACE inhibitor   (Altace or Piramil/Ramipril)   1. Other (description) 2. None | |  |
| 137 | What medication should one give someone with blood pressure of >160/100?  Yes------1  No------2 | 1. Anti depressant   (Frengit or Meltix/Flupentixol Meletracin)   1. Sedative/Sleeping Pill   (Bupam/Bomazepam; Clobazam/Frizium  Diazepam/Ezium)   1. Beta Blocker   (Teneloc/Atenolol; Indever/Propanolol)   1. Calcium channel blocker   (Amdocol/Amlodipine; Nipin/Nefidipine   1. Diuretic   (Lasix/Furosemide; Dezide/HZT)   1. Losartan Potassium   (Osartil)   1. ACE inhibitor   (Altace or Piramil/Ramipril)   1. Other (description) 2. None | |  |
|  |  |  | |  |
| 138 | How long should one usually recommend that a hypertension patient take medications? | 0-7 Days 1  1-4 Weeks 2  1-12 months 3  1-2 Years 4  >2 years 5  Forever 6  Until free of symptoms 7  Until blood pressure is normal 8 | |  |
| 139 | Do you refer hypertension patients? | Nevcr 1  Rarely 2  Sometimes 3  Often 4  Always 5 | |  |
| 140 | Where do you refer hypertension patients  Yes------1  No------2 | 1. MBBS 2. MBBS Specialist 3. Cardiologist 4. Respiratory Disease Hospital 5. Government Hospital 6. Village Doctor 7. Drug Seller 8. Diagnostic Center 9. National Heart Foundation 10. Other (description) 11. None | |  |
| 141 | Where do you most commonly refer hypertension patients?  Description: | MBBS 1  MBBS Specialist 2  Cardiologist 3  Respiratory Disease Hospital 4  Government Hospital 5  Village Doctor 6  Drug Seller 7  Diagnostic Center 8  National Heart Foundation 9  Other (description) 10 | |  |
|  |  |  | |  |
| 142 | At what blood pressure should otherwise healthy adults with hypertension patients go directly to an MBBS doctor for treatment?  If the patient is 18 to 60 years old?  If the patient is more than 60 years old?  1) Systolic  2)Diastolic | 1) 2)  >60 a) /    <60 b) / | |  |
